# Dynamic monitoring and adaptive algorithm optimization of fatigue recovery in track and field athletes based on inertial measurement unit sensor data

**DOI:** 10.3389/fphys.2026.1919336

**Published:** 2026-10-06

**Authors:** Jian Xu, Junwei Bai

**Affiliations:** China Academy of Art, Hangzhou, China

**Keywords:** adaptive modelling, collegiate sprinters, inertial measurement unit, recovery monitoring, sprint fatigue, sprint recovery adaptation

## Abstract

**Introduction:**

Sprint recovery monitoring requires individualized assessment because movement mechanics return toward baseline at different rates following repeated high-intensity loading. This study developed Sprint Recovery Adaptation (SRA), an adaptive framework for individualized sprint-kinematic recovery monitoring in 100 m and 200 m sprinters.

**Methods:**

A repeated-measures field algorithm-validation design included 124 Chinese collegiate sprinters who completed baseline testing, repeated 60 m fatigue-induction sprints, and recovery assessments immediately post-fatigue and at 5, 10, 15, and 20 min. SRA integrated inertial measurement unit (IMU)-derived acceleration consistency, angular-velocity control, trunk-pelvis stability, limb symmetry, individualized baseline normalization, and temporal updating. Recovery categories were defined according to predefined testing intervals and individualized mechanical deviation criteria. Because no independent physiological reference measure was included, interpretation was restricted to sprint-kinematic recovery. An independent endurance-running IMU dataset was additionally used for cross-task feature-generalization testing.

**Results:**

The Recovery Index increased from 54.20 ± 8.30 immediately post-fatigue to 86.80 ± 6.40 at 20 min. Athlete-level held-out validation yielded an accuracy of 0.864, an F1-score of 0.861, and an area under the curve of 0.917, with SRA outperforming the strongest fixed comparison model. Ablation analysis identified individualized baseline normalization and temporal updating as major contributors to classification performance. Cross-task testing using the independent endurance-running IMU dataset supported the generalizability of the feature-processing pipeline.

**Discussion:**

SRA provides a feasible approach for short-term, individualized monitoring of post-fatigue sprint-mechanics recovery. Its adaptive use of athlete-specific baselines and temporal updating appears to improve recovery-state classification compared with fixed models. However, the framework should be interpreted as a measure of kinematic recovery rather than physiological recovery, and further validation against independent physiological and performance-based reference measures is warranted.

## 1 Introduction

Sprint training is a high-intensity athletic process designed to develop acceleration, maximal velocity, stride efficiency, and repeated force production in short-distance events. Neuromuscular fatigue is an exercise-induced reduction in voluntary force capacity arising from central and peripheral mechanisms (Alba-Jiménez et al., 2022), while fatigue recovery describes restoration of performance, physiological, psychological, and biochemical status after fatigue-related decline (Bestwick-Stevenson et al., 2022). This process must be read through sprint biomechanics, where stride length, stride frequency, force production, ground-reaction-force orientation, and mechanical efficiency determine performance quality (Pietraszewski et al., 2025). An inertial measurement unit (IMU) supports field assessment by collecting acceleration and angular velocity, from which orientation estimates and kinematic variables are derived (Liang et al., 2023). Fixed models may miss nonlinear athlete-specific recovery patterns, so adaptive modeling is required to represent changing fitness–fatigue responses (Imbach et al., 2022).

Fatigue detection, fatigue-stage classification, and recovery-state estimation represent distinct analytical tasks. Fatigue detection identifies exercise-associated deterioration from a rested condition, while fatigue-stage classification differentiates the progression or severity of that deterioration across observations. Recovery-state estimation concerns the subsequent trajectory through which performance and movement characteristics approach an established rested reference. In the present study, recovery is operationalized as sprint-kinematic recovery, defined as the progressive return of sprint performance and inertial measurement unit (IMU)-derived movement characteristics toward each athlete’s rested baseline profile. Sprint time, countermovement-jump performance, perceived exertion, acceleration consistency, angular-velocity control, trunk-pelvis stability, and limb symmetry provide complementary indicators of this short-term recovery trajectory. Electrophysiological, biochemical, and direct muscle-function measurements were not collected; consequently, the measured construct is confined to movement-mechanical and performance-related recovery following repeated sprint loading.

Current evidence supports wearable fatigue monitoring but does not establish a sprint-specific adaptive recovery framework. Recent biomechanical and computational evidence further supports the use of wearable signals for characterizing fatigue-related mechanical change. Xu et al. (2025) integrated wearable inertial signals, musculoskeletal information, and deep learning to estimate ligament loading and fatigue-failure mechanisms during repeated movement, demonstrating that sensor-derived features can represent mechanically meaningful responses to cumulative loading. Zhou et al. (2026) extended this approach through multimodal structural and movement information for tissue-specific running fatigue modeling, linking individualized mechanical characteristics with cumulative loading and fatigue-related risk. These developments strengthen the biomechanical basis for sensor-based fatigue assessment, although their outcomes concern tissue loading and fatigue-related mechanical risk and leave short-term post-sprint movement recovery insufficiently characterized.

Wearable systems identify fatigue through posture, acceleration, coordination, and technique changes, although broad reviews rarely define recovery states for maximal sprinting (Chen et al., 2025). Artificial intelligence (AI) improves fatigue recognition when wearable time-series data use careful feature selection and personalization, but reliability still depends on sport-specific validation (Kakhi et al., 2025). Inertial-sensor running studies identify stride time, stride frequency, impact acceleration, and spatiotemporal variability as fatigue-sensitive variables, yet distance-running protocols cannot represent sprint recovery directly (McConnochie et al., 2025). Time-series modeling shows that IMU data can detect subtle movement change before visible fatigue (Biró et al., 2023), and repeated-sprint testing confirms declines in sprint decrement, jump performance, and neuromuscular function (Armada-Cortés et al., 2022). Missing elements include recovery labels, feature thresholds, athlete-specific updating, and validation against sprint recovery states.

Repeated-sprint research further indicates that different performance and neuromuscular indicators recover at different rates after high-intensity loading. Pimenta et al. (2024) identified progressive alterations in knee-flexor peak torque and neuromuscular parameters during repeated sprinting, demonstrating substantial peripheral and performance-related responses to repeated maximal efforts. Yang et al. (2026) similarly reported immediate reductions in countermovement-jump and isometric-force performance after repeated-sprint training, with residual neuromuscular and perceptual changes persisting across subsequent recovery assessments. Such findings support multidimensional monitoring of post-sprint recovery because restoration of sprint performance, movement control, and neuromuscular indicators may occur along different temporal trajectories.

Sprint-specific studies justify the proposed model inputs but expose the methodological gap. IMU hip-flexion power relates to sprint performance qualities (Nagahara and Murata, 2020), shoe-mounted sensors quantify outdoor foot-speed mechanics (Pla et al., 2024), and overground IMU systems validly register lower-limb and trunk kinematics against high-speed video (de Ruiter et al., 2024). Applied sprint profiling can integrate kinematic and force–velocity variables (Wolski et al., 2025), although calibration choices affect segment-level accuracy (Ekdahl et al., 2023). Repeated-sprint analysis shows distinct fatigue-adaptation clusters across athletes (Romero et al., 2022). Evidence on sprint-start mechanics (Li et al., 2024) and collegiate sprinter neuromuscular profiles (Li et al., 2026) indicates that collegiate sprinters require population-specific modeling because training background, event specialization, and technical profile may influence post-fatigue movement responses and recovery trajectories. Validated adaptive IMU models for individualized recovery-state classification in 100 m and 200 m sprinters remain insufficiently developed. The specific methodological gap concerns the integration of athlete-specific baseline referencing, multiple sprint-biomechanical feature domains, temporal recovery information, and athlete-level validation within a single framework for estimating return toward rested sprint mechanics. Sprint Recovery Adaptation (SRA) is developed to address this gap through individualized sprint-kinematic recovery estimation following repeated sprint loading.

### 1.1 IMU evidence for running-fatigue monitoring

IMU running-fatigue evidence shows measurable biomechanical deterioration, but most protocols reflect endurance pacing instead of sprint-specific recovery. Zhang et al. (2025) used rearfoot, shank, and pelvis IMUs during an outdoor marathon, showing slower speed, longer stance/stride time, reduced propulsion acceleration, altered rearfoot rotation, shank tilt, and pelvic acceleration across 5 km intervals. Its strength is continuous field monitoring, yet prolonged aerobic-metabolic fatigue differs from repeated maximal acceleration fatigue in 100 m and 200 m athletes. Mitschke et al. (2025) assessed a half-marathon with IMU, heart-rate, and perceived-exertion data, finding higher tibial acceleration, foot angular velocity, and rearfoot eversion velocity during fatigue. However, it detected fatigue during prolonged running without tracking return-to-baseline mechanics after exercise. Dimmick et al. (2023) combined inertial signals with musculoskeletal simulation, revealing individualized torso-pelvis adaptations, although modeled mechanics may miss real-time sprint asymmetry, acceleration loss, and recovery dynamics. No clear recovery labels, thresholds, or post-fatigue intervals are established.

Sprint-fatigue monitoring depends on whether inertial measurement unit signals remain sensitive and reliable across the specific running task and biomechanical domain assessed. Horsley et al. (2024) found that thoracic-worn accelerometers detected fatigue-related changes in vertical stiffness following repeated sprinting, indicating that trunk acceleration can capture acute alterations in sprint mechanics. Dimmick et al. (2022) reported good-to-excellent between-day reliability for multiple center-of-mass inertial measurement unit features during fatiguing running and showed that these features remained responsive to fatigue across controlled running intensities. Marotta et al. (2022) identified tibial and sacral acceleration as recurrent fatigue-sensitive variables across cyclical lower-limb exercise studies, while also documenting substantial variation in fatigue criteria, sensor configurations, and analytical procedures. These findings support acceleration-derived fatigue assessment and highlight the importance of standardized sprint protocols, athlete-specific reference profiles, and explicit criteria for interpreting movement changes during post-fatigue recovery.

### 1.2 Adaptive algorithms for fatigue-state prediction

Adaptive fatigue algorithms show predictive promise, but fatigue detection, stage classification, and recovery prediction remain methodologically different tasks. Marotta et al. (2021) used track-based IMU data, random forest classification, feature selection, and leave-one-subject-out validation during repeated 400 m running, finding that tibia-thigh sensor sets captured angular velocity, acceleration magnitude, and gait variability with practical accuracy. However, perceived-exertion labels may introduce misclassification and weaken physiological interpretation. Chang et al. (2023) used IMU time series, heart rate, perceived exertion, LSTM, CNN, attention-based LSTM, and LSTM-CNN models to classify pre-fatigue, mid-fatigue, and post-fatigue states. The high classification performance supports temporal modeling, but healthy runners differ from trained sprinters in force production, acceleration mechanics, and recovery demands. Biró et al. (2026) showed that personalized random forest and one-class SVM models outperformed population models, yet limited external validation, feature robustness testing, and recovery labels constrain generalizable athlete-specific prediction.

Adaptive fatigue modeling increasingly relies on multidimensional movement representation, multimodal sensing, and independently defined reference labels to improve state discrimination. Rousseau et al. (2024) used inertial measurement unit and force-derived movement data with adversarial autoencoders to represent fatigue-associated motor-control changes within a multidimensional latent space, demonstrating that fatigue can be expressed through distributed changes in movement patterns. Hwang et al. (2025) integrated surface electromyography (sEMG) and inertial measurement unit signals within a convolutional neural network–long short-term memory–attention architecture and evaluated fatigue recognition across participants, emphasizing multimodal feature integration and participant-independent validation. Manoharan et al. (2026) used sEMG-derived physiological fatigue markers to generate reference labels for inertial measurement unit-based random forest and neural-network classifiers, strengthening separation between label definition and subsequent inertial-signal prediction. Existing approaches therefore provide strong foundations in feature representation, multimodal integration, and reference-label design, while sprint-specific post-fatigue recovery estimation still lacks athlete-specific baseline normalization, temporal updating across recovery intervals, multi-domain movement integration, and athlete-level validation within a single analytical framework.

### 1.3 Analytical framework

Endurance-based IMU studies identify fatigue-sensitive acceleration, angular-velocity, impact-loading, and trunk-pelvis variables, yet they do not define sprint-specific recovery labels or return-to-baseline thresholds. Algorithmic studies show that personalized models can outperform fixed models, yet most classify fatigue presence or fatigue stage without modeling recovery progression after maximal sprint work. The study develops Sprint Recovery Adaptation (SRA) to address these issues (**Figure 1**). SRA links three components: IMU feature extraction, individualized fatigue-response profiling, and adaptive recovery-state estimation. Acceleration consistency is extracted from stride-to-stride acceleration variation; angular-velocity control from segment rotational patterns; trunk-pelvis stability from lumbar and pelvic signal variability; and recovery trajectory from the degree of movement return toward the athlete-specific rested profile.

**Figure 1:**
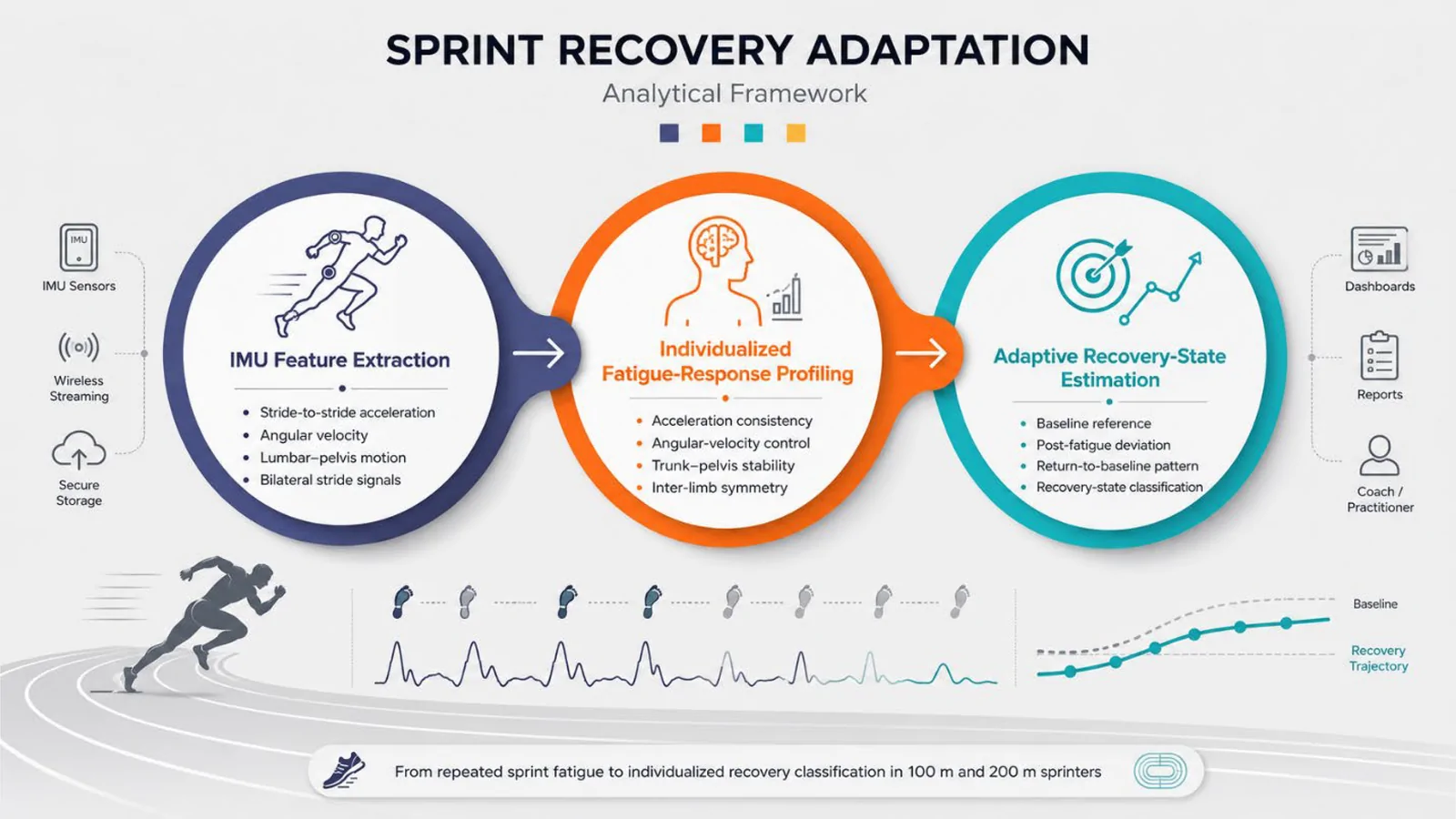
Sprint recovery adaptation analytical framework showing IMU feature extraction, individualized fatigue-response profiling, and adaptive recovery-state estimation for classifying return-to-baseline recovery patterns in 100 m and 200 m sprinters.

Methodologically, SRA integrates four operations within one athlete-level recovery pipeline: individualized normalization against the rested sprint profile, integration of acceleration consistency, angular-velocity control, trunk-pelvis stability, and limb-symmetry information, temporal updating across predefined post-fatigue intervals, and joint estimation of interval-linked recovery categories with a continuous Recovery Index. This structure defines SRA as an adaptive model of individualized sprint-kinematic recovery progression and separates its analytical target from general fatigue detection and physiological neuromuscular recovery assessment.

SRA advances IMU-derived sprint monitoring from isolated fatigue classification to athlete-specific estimation of post-fatigue movement progression. Its methodological contribution lies in combining individualized baseline normalization, multi-domain sprint-mechanics integration, and temporal updating within the same recovery-estimation procedure. Recovery categories represent interval-linked stages of the observed sprint-kinematic trajectory, while the Recovery Index quantifies continuous movement return toward the athlete-specific baseline.

### 1.4 Objectives

The study aims to (i) identify inertial measurement unit features associated with post-sprint fatigue and subsequent return toward baseline movement mechanics, (ii) develop SRA for individualized sprint-kinematic recovery estimation across predefined post-fatigue intervals, and (iii) compare SRA with fixed machine-learning models using athlete-level held-out validation.

The study is guided by the following research questions. RQ1: Which inertial measurement unit sprint-biomechanical features are most sensitive to post-fatigue change and subsequent return toward baseline? RQ2: Does adaptive modeling improve individualized sprint-kinematic recovery estimation compared with fixed machine-learning models? RQ3: How effectively does SRA characterize individual recovery progression across immediate post-fatigue, 5 min, 10 min, 15 min, and 20 min assessments?

Based on the study objectives and research questions, the following hypotheses are proposed. H1: Stride, acceleration, angular-velocity, stability, and symmetry features will change following repeated sprint loading and progressively approach athlete-specific baseline values during recovery. H2: SRA will achieve stronger held-out athlete recovery-estimation performance than fixed machine-learning models. H3: Recovery Index values will increase across predefined post-fatigue intervals as sprint-kinematic features progressively approach athlete-specific rested profiles.

## 2 Methods

### 2.1 Research design

The study adopted a repeated-measures field-based algorithm-validation design with Chinese collegiate 100 m and 200 m sprinters. Each athlete completed rested baseline, immediate post-fatigue, and fixed recovery-interval assessments after repeated sprint loading. The rested baseline condition functioned as the within-athlete control condition because recovery estimates were interpreted against each athlete’s own non-fatigued sprint profile. SRA was implemented as the main adaptive algorithmic condition, using IMU-derived acceleration, angular-velocity, stability, symmetry, and return-to-baseline features. Fixed non-adaptive machine-learning models and a non-adaptive SRA variant served as algorithmic control conditions for performance comparison. The non-adaptive SRA control retained the same IMU feature domains, preprocessing pipeline, classifier architecture, training observations, and hyperparameters as the full SRA model. Its athlete-specific baseline vector was fixed at the mean of the three valid baseline trials, and no temporal state update was applied after the immediate post-fatigue observation. Consequently, differences between full SRA and non-adaptive SRA isolated the contribution of baseline adaptation and temporal recovery updating while holding feature information and classifier capacity constant.

This study involved human participants and was conducted under IRB approval protocol IRB-SPR2026-041. Written informed consent was obtained before participation, and voluntary participation, withdrawal rights, pre-test health screening, supervised sprint testing, anonymized data handling, and predefined safety stopping criteria were maintained throughout the study. The study was conducted in Chinese university athletics setting using a standard synthetic sprint track with consistent lane allocation, warm-up routine, footwear condition, recovery area, and testing schedule. The unit of analysis was the athlete-session and stride-level IMU signal segment collected across baseline, post-fatigue, and recovery intervals.

### 2.2 Participants

Participants were Chinese collegiate short-distance sprinters specializing in the 100 m and 200 m events and actively training within university athletics programs. They represented a trained athletic population with regular exposure to acceleration drills, maximal-velocity running, sprint-specific strength work, and repeated high-intensity track sessions. This population was selected because sprint fatigue and recovery are strongly reflected in stride rhythm, lower-limb acceleration, angular-velocity control, trunk-pelvis stability, and return-to-baseline movement mechanics after repeated sprint loading.

#### 2.2.1 Recruitment

Participants were approached through athletics departments and sprint-team coaches at Chinese universities. Recruitment notices described the testing schedule, sprint-fatigue protocol, IMU placement, recovery monitoring, data confidentiality, and withdrawal rights. A prior power analysis was conducted using G*Power 3.1 with α = 0.05, statistical power = 0.80, a moderate repeated-measures effect assumption, and five fatigue-recovery measurement points (Faul et al., 2007). The minimum target was increased to support athlete-level model validation, sex-event stratification, and anticipated attrition. A total of 178 sprinters were approached; 164 agreed to screening, 14 were unavailable for testing, 12 did not meet sprint-event specialization, 10 had recent lower-limb health problems, 7 had positive health-screening responses without medical clearance, 5 had incomplete baseline records, and 6 withdrew before fatigue testing, leaving 124 participants for analysis.

#### 2.2.2 Eligibility criteria

Inclusion criteria were (i) age 18–24 years; (ii) registered Chinese collegiate athletics-team membership; (iii) primary specialization in 100 m or 200 m sprint events; (iv) ≥2 years of structured sprint training; (v) ≥4 sprint-training sessions per week during the preceding 3 months; (vi) current training availability confirmed by the team coach; (vii) no positive Physical Activity Readiness Questionnaire for Everyone response requiring medical restriction, or documented medical clearance when a positive response occurred (Warburton et al., 2011); and (viii) baseline rating of perceived exertion ≤11 on the Borg 6–20 scale before sprint testing (Borg, 1998). Exclusion criteria were (i) lower-limb surgery within 6 months; (ii) current hamstring, quadriceps, calf, ankle, hip, or lumbar injury; (iii) Oslo Sports Trauma Research Center Questionnaire on Health Problems response indicating substantial training reduction, performance reduction, or inability to participate during the previous 7 days (Clarsen et al., 2020, 2014); (iv) cardiovascular, neurological, vestibular, or respiratory contraindication to maximal sprinting; and (v) incomplete IMU recording during baseline or post-fatigue trials.

#### 2.2.3 Stratification and bias

Participants were stratified by sprint event, sex, and performance level before athlete-level allocation into the SRA development cohort and held-out validation cohort. The SRA development cohort was used for feature extraction, individualized fatigue-response profiling, adaptive model training, and internal cross-validation, while the held-out validation cohort was reserved for final recovery-state testing. The within-athlete rested baseline condition was retained as the experimental control condition for interpreting post-fatigue and recovery mechanics. Bias was minimized through standardized warm-up, identical sprint-fatigue protocol, fixed recovery intervals, consistent lane allocation, same sensor-placement map, blinded recovery-state labeling, athlete-level data splitting, and separate model testing on unseen participants.

The 124 athletes were allocated at an approximately 80:20 ratio using stratified athlete-level randomization with a fixed seed of 2026. The development cohort comprised 99 athletes, including 53–100 m and 46–200 m sprinters, 57 males and 42 females, and 33 athletes in each sex- and event-specific performance tertile. The held-out cohort comprised 25 athletes, including 13–100 m and 12–200 m sprinters, 14 males and 11 females, with 8, 9, and 8 athletes in the lower, middle, and higher performance tertiles, respectively. Training-age strata included 54 athletes with 2–4 years and 45 with >4 years in the development cohort and 14 and 11 athletes, respectively, in the held-out cohort. All observations from a given athlete were assigned to one cohort, preventing baseline, post-fatigue, or recovery observations from the same athlete from appearing in both development and held-out datasets. Feature scaling, feature selection, principal-component estimation, classifier fitting, and hyperparameter selection were completed after athlete-level allocation. Parameters estimated from the development cohort were frozen before the held-out cohort was accessed for final performance evaluation.

#### 2.2.4 Sensor configuration and calibration

IMU data were collected using six wireless Ultium Motion sensors (Noraxon USA Inc., Scottsdale, AZ, USA) sampled at 200 Hz, with accelerometer range set to ±16 g and gyroscope range set to ±2000°/s. Sensors were fixed at the fifth lumbar vertebra, sacral pelvis, bilateral distal shanks 5 cm above the medial malleolus, and bilateral dorsal feet over the second metatarsal. Each site was cleaned with 70% isopropyl alcohol, dried for 60 s, marked with skin-safe ink, and secured using adhesive interfaces, elastic cohesive wrap, and non-restrictive sports tape (**Figure 2**).

**Figure 2:**
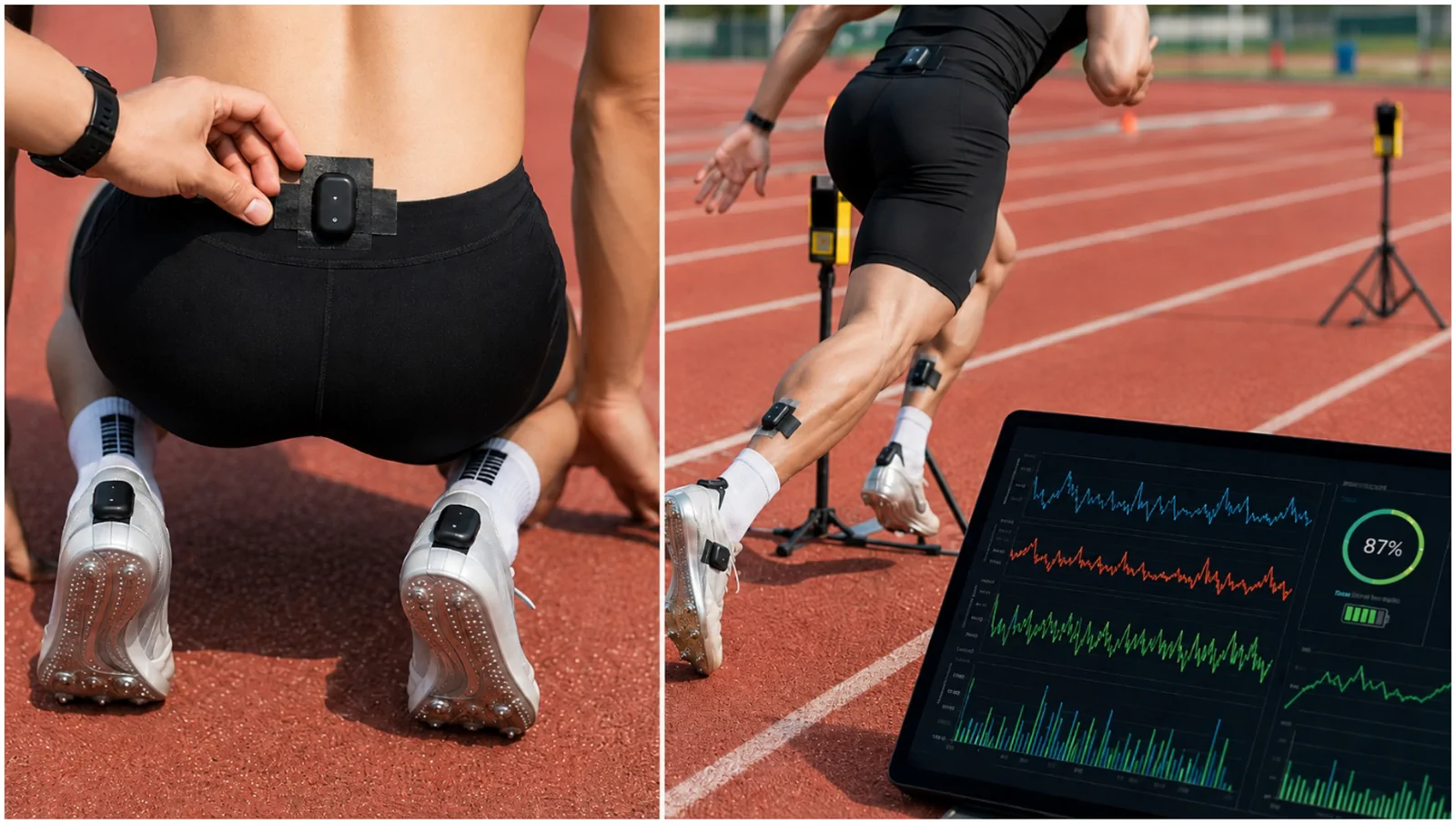
Experimental implementation of IMU-based sprint recovery monitoring showing lumbar, pelvic, bilateral distal-shank, and bilateral dorsal-foot sensor placement and field-based sprint testing with synchronized stride-signal acquisition across baseline, fatigue, and recovery assessments.

Before each testing phase, sensors were paired with the acquisition hub, battery status was confirmed at ≥90%, and a 30 s standing trial was recorded to check signal stability. Static calibration was performed for 10 s in upright anatomical posture, followed by 20 m progressive jogging, 3 submaximal accelerations, and 3 countermovement jumps to confirm segment alignment and sensor fixation. Synchronization was checked using one simultaneous bilateral heel strike and one vertical jump before each sprint block. Trials were accepted only when onset mismatch was ≤10 ms and no sensor displacement or signal dropout occurred.

### 2.3 SRA protocol

SRA was implemented as a three-stage protocol linking IMU feature extraction, individualized fatigue-response profiling, and adaptive recovery-state estimation. First, stride-level IMU signals were extracted from lumbar, pelvis, bilateral shank, and bilateral foot sensors during baseline, post-fatigue, and recovery trials. Second, each athlete’s rested baseline profile was used as the within-athlete control profile, and all post-fatigue features were converted into percentage deviation from that athlete’s baseline mean. Third, recovery-state estimation classified each trial into five classes: rested baseline, acute fatigue, early recovery, intermediate recovery, and late recovery. Acceleration consistency was calculated as the coefficient of variation of stride-to-stride resultant acceleration; angular-velocity control was calculated from peak and variability values of foot, shank, pelvis, and lumbar rotation; trunk-pelvis stability was calculated from lumbar and pelvic acceleration variability; inter-limb symmetry was calculated from left–right shank and foot differences; and recovery status was calculated from the degree of return toward the athlete’s own baseline movement profile (**Supplementary Table S1**).

#### 2.3.1 Baseline and fatigue-induction protocol

Athletes completed a standardized 15 min warm-up consisting of 5 min light jogging, 5 min dynamic mobility, three 30 m progressive accelerations at approximately 60%, 75%, and 90% effort, and 3 min seated rest before baseline testing. Sprint time was recorded using Witty timing gates (Microgate S.r.l., Bolzano, Italy) placed at 0 m, 30 m, and 60 m. Baseline testing consisted of three maximal 60 m sprints from a standing start, separated by 4 min passive recovery, with IMU data recorded throughout each sprint. The fastest valid sprint and the mean of three valid trials were retained for baseline profiling.

For recovery analyses, only the matched 0–30 m acceleration phase of each baseline 60 m sprint was used. The 30 m split and corresponding IMU samples were extracted from all three baseline trials using the same start event, stride-segmentation rules, filtering pipeline, and feature definitions applied to the subsequent 30 m recovery trials. The athlete-specific reference vector was calculated as the mean of the three baseline 0–30 m feature vectors. The full 60 m baseline time remained a descriptive sprint-performance variable and was excluded from direct comparison with the 30 m recovery trials. Recovery trials used one 30 m effort at each interval to limit additional fatigue; each trial was summarized from all valid strides within the 0–30 m segment using the same stride-level aggregation procedure used for the baseline segments.

Fatigue induction used repeated 60 m maximal sprints from a standing start, with 90 s passive recovery between sprints. The protocol continued until the athlete reached at least one predefined fatigue criterion: sprint time increased by ≥5% compared with baseline, countermovement-jump height decreased by ≥10% compared with baseline, rating of perceived exertion reached ≥17 on the Borg 6–20 scale, or sprint mechanics showed unsafe deterioration confirmed by the supervising coach. Countermovement-jump height was recorded using OptoJump Next (Microgate S.r.l., Bolzano, Italy), with three jumps performed before fatigue induction and immediately after the final sprint. Athletes completed a minimum of 6 and a maximum of 10 fatigue sprints to ensure comparable loading while preventing excessive fatigue exposure.

Fatigue dose was quantified for each athlete using the number of completed 60 m repetitions, cumulative sprint time, and percentage slowing of the final sprint relative to baseline. Sprint count was retained as a continuous centered covariate in the primary recovery models, with a sensitivity analysis grouping athletes into 6–7, 8, and 9–10 repetition strata. This procedure accounted for variation in exposure caused by athletes meeting the prespecified stopping criteria at different repetitions.

#### 2.3.2 Recovery monitoring procedure

Recovery monitoring began within 60 s after the final fatigue sprint. Athletes remained in the same recovery area and completed passive standing or seated recovery without stretching, massage, fluid loading, or coaching feedback that could change recovery behavior. IMU signals, sprint time, countermovement-jump height, and rating of perceived exertion were recorded at immediate post-fatigue, 5 min, 10 min, 15 min, and 20 min. At each recovery interval, athletes completed one 30 m maximal-effort sprint-mechanics trial from a standing start using the same effort instruction and 0–30 m acceleration-phase definition applied to baseline testing and one countermovement-jump set of three attempts. The 30 m distance was used during recovery monitoring to reduce additional fatigue while preserving acceleration-phase mechanics relevant to 100 m and 200 m sprint performance.

Recovery-state labels were prespecified from the experimental schedule before model-feature extraction and were independent of the IMU predictors used by SRA. Rested baseline represented the pre-fatigue assessment, acute fatigue the immediate post-fatigue assessment, early recovery the 5 min assessment, intermediate recovery the 10- and 15-min assessments, and late recovery the 20 min assessment. Testing interval itself was not supplied to the classifier as a predictor. Sprint time, countermovement-jump height, and rating of perceived exertion were excluded from the SRA predictor matrix and were retained as independent criterion variables for secondary mechanical-recovery verification. Mechanical recovery was defined as 30 m sprint time within +5% of the athlete-specific baseline mean and countermovement-jump height within −10% of baseline; observations failing one criterion were categorized as partial mechanical recovery, and observations failing both criteria were categorized as fatigue-dominant. Two trained assessors independently verified protocol labels from timestamped acquisition records and independently classified the secondary mechanical-recovery criterion while blinded to SRA predictions. Inter-rater agreement was κ = 0.88, with disagreements resolved by consensus before analysis.

#### 2.3.3 SRA computation and adaptive updating

Raw acceleration and angular-velocity signals were low-pass filtered using a fourth-order zero-phase Butterworth filter with a 20 Hz cutoff. Feature extraction produced four athlete-level domains at each interval: acceleration consistency (A), angular-velocity control (G), trunk-pelvis stability (T), and inter-limb symmetry (L). For athlete i, feature j, and recovery interval t, proportional deviation from the current athlete-specific reference was calculated as, ${\text{d}}_{\text{ijt}}=\frac{\left|{\text{x}}_{\text{ijt}}-{\text{b}}_{\text{ijt}}\right|}{\left|{\text{b}}_{\text{ijt}}\right|+\epsilon },$ where x_ijt_ is the observed feature value, b_ijt_ is the athlete-specific reference value, and $\epsilon =10^{-6}$ prevents division by zero. Deviations were truncated at 0.30 to limit the influence of extreme observations, and domain recovery scores were calculated as, ${\text{S}}_{\text{ijt}}=100\left[1-\text{min}\left(\frac{{\text{d}}_{\text{ijt}}}{0.30},1\right)\right].$ The Recovery Index was calculated as ${\text{RI}}_{\text{it}}=0.32{\text{S}}_{\text{Ait}}+0.26{\text{S}}_{\text{Git}}+0.23{\text{S}}_{\text{Tit}}+0.19{\text{S}}_{\text{Lit}}.$ Feature extraction produced four athlete-level domains at each interval: acceleration consistency, angular-velocity control, trunk-pelvis stability, and inter-limb symmetry, which were subsequently integrated through Recovery Index computation, adaptive baseline updating, temporal updating, classification, cross-validation, and held-out athlete evaluation (**Supplementary Figure S1**).

The four weights were obtained from the normalized absolute standardized coefficients of the development-cohort recovery model and were frozen before held-out evaluation. The ±5% movement threshold was specified as a conservative dead-band for athlete-level return toward baseline; deviations ≤5% were treated as mechanically close to the rested reference. Sensitivity analyses using ±3% and ±7% thresholds were specified to examine dependence on this criterion. The initial baseline vector b_i0_ was the mean feature vector from the three matched 0–30 m baseline segments. During recovery, the baseline reference remained unchanged until the independent sprint-time and countermovement-jump criteria indicated mechanical recovery. After that criterion was satisfied, the reference for the subsequent interval was updated using, ${\text{b}}_{\text{ijt}}=0.80{\text{b}}_{\text{ij},\text{t}-1}+0.20{\text{x}}_{\text{ij},\text{t}-1}.$ This restricted update prevented strongly fatigued observations from shifting the athlete-specific reference. The non-adaptive SRA variant retained ${\text{b}}_{\text{ijt}}={\text{b}}_{\text{ij}0}$ for all intervals.

Temporal information was incorporated using an exponentially weighted feature state, ${\text{z}}_{\text{ijt}}=0.70{\text{d}}_{\text{ijt}}+0.30{\text{z}}_{\text{ij},\text{t}-1},$ with ${\text{z}}_{\text{ij}1}={\text{d}}_{\text{ij}1}$ at the immediate post-fatigue assessment. Current deviation, temporally updated deviation, change from the preceding interval, and the four domain recovery scores formed the adaptive classifier input. Recovery-state classification used a five-class gradient-boosted decision-tree classifier with softmax output. Hyperparameters were selected by five-fold group cross-validation within the 99-athlete development cohort and fixed at 250 boosting iterations, maximum tree depth = 3, learning rate = 0.05, subsampling fraction = 0.80, column-sampling fraction = 0.80, minimum child weight = 3, and L2 regularization parameter = 1.0. Early stopping was applied after 25 iterations without improvement in validation log loss. All folds were grouped by athlete identifier.

### 2.4 Measures and outcomes

#### 2.4.1 Primary outcomes

Recovery State was the primary categorical outcome and was assigned at five time points: rested baseline, immediate post-fatigue, 5 min, 10 min, 15 min, and 20 min. At baseline, athletes completed three maximal 60 m sprints; after fatigue and during recovery, athletes completed one standardized 30 m sprint at each interval to reduce additional load while retaining acceleration mechanics. Recovery State was defined prospectively from the fixed experimental assessment schedule, and testing interval was excluded from the SRA predictor matrix. Sprint time and countermovement-jump change were retained for secondary mechanical-recovery verification and were not used as SRA predictors. Protocol-label integrity and secondary mechanical-recovery classification were independently checked by two trained assessors blinded to model output (κ = 0.88).

Recovery Index was the primary continuous outcome and represented each athlete’s return toward the rested baseline movement profile. The index was calculated at immediate post-fatigue, 5 min, 10 min, 15 min, and 20 min using the weighted deviation of acceleration consistency, angular-velocity control, trunk-pelvis stability, and inter-limb symmetry from each athlete’s own baseline mean. A value closer to 100 indicated stronger return to baseline, while lower values indicated persistent fatigue-related deviation. Three baseline sprint trials were averaged to form the athlete-specific reference profile, and each recovery trial was compared against that reference.

#### 2.4.2 Secondary outcomes

Sprint Time was measured using Witty timing gates (Microgate S.r.l., Bolzano, Italy) positioned at 0 m, 30 m, and 60 m during baseline testing and at 0 m and 30 m during recovery monitoring. Three 60 m baseline trials were recorded, and one 30 m sprint was recorded at each post-fatigue and recovery interval. Jump Height was measured using OptoJump Next (Microgate S.r.l., Bolzano, Italy), with three countermovement jumps recorded at baseline, immediately after fatigue, and each recovery interval; the best valid jump was retained. Stride Variability was extracted from foot-sensor stride intervals as the coefficient of variation across valid strides.

Stride Frequency was calculated as the number of complete strides per second during each sprint trial. Acceleration Consistency was calculated from stride-to-stride resultant acceleration variation across foot, shank, pelvis, and lumbar sensors. Angular Control was derived from peak and variability values of foot, shank, pelvis, and lumbar angular velocity. Trunk Stability was derived from lumbar and pelvic acceleration variability. Limb Symmetry was calculated from left–right percentage differences in matched foot and shank features. Exertion Score was recorded immediately after each sprint block using the Borg 6–20 rating of perceived exertion scale, with scores ≥17 used as one fatigue-confirmation criterion (Borg, 1998).

#### 2.4.3 Model comparators, ablations, and validation

Ablation analysis tested whether SRA performance depended on its full feature and adaptation structure. The full SRA model was first trained using acceleration consistency, angular control, trunk stability, limb symmetry, individualized baseline updating, and temporal recovery updating. Six reduced variants were then created by removing one component at a time: no acceleration features, no angular-velocity features, no trunk-pelvis features, no limb-symmetry features, no individualized baseline updating, and no temporal recovery updating. Each ablation used the same athlete-level training split, recovery labels, and validation procedure as the full model, so performance change reflected the removed component rather than sampling differences.

Baseline algorithms were selected from recent IMU running-fatigue studies with comparable wearable-sensor inputs and fatigue-state prediction aims. Random forest was included because track-based IMU fatigue work used feature-selected random forest models with leave-one-subject-out validation to classify running fatigue (Marotta et al., 2021). Long short-term memory and convolutional neural network–long short-term memory models were included because IMU time-series fatigue-stage classification showed that temporal and hybrid deep-learning architectures captured fatigue patterns from raw running signals (Chang et al., 2023). One-class support vector machine was included as a personalized abnormal-state detector trained on each athlete’s baseline mechanics, allowing fatigue and incomplete recovery to be interpreted as deviation from the non-fatigued reference profile.

Cross-task feature-generalization testing used the publicly available Multivariate Time Series Data of Fatigued and Non-Fatigued Running from IMUs dataset (Kathirgamanathan et al., 2023). The endurance-running dataset was used exclusively to test whether the SRA feature-extraction and fatigue-discrimination pipeline retained discriminatory information in an independent running task. It was not used to evaluate sprint-recovery categories, Recovery Index estimation, temporal recovery updating, or athlete-specific post-sprint recovery trajectories. Sensor channels were harmonized to the corresponding lower-limb and trunk signals, recordings were resampled to 200 Hz, and the feature transformations learned from the SRA development cohort were applied without refitting. Performance was evaluated only for binary non-fatigued versus fatigued discrimination and is reported as cross-task feature generalization.

#### 2.4.4 Subgroup analysis

Subgroup analysis examined whether SRA recovery estimation differed across athlete characteristics that were directly relevant to sprint performance. Event Specialty compared 100 m and 200 m sprinters because the 200 m event involves longer speed-endurance demand and may produce slower return-to-baseline mechanics than the 100 m event. Sex compared male and female athletes because force output, stride mechanics, and fatigue response may differ across groups. Performance Level compared higher-performing and lower-performing collegiate sprinters using sex-specific and event-specific personal-best tertiles recorded before testing. Training Age compared athletes with 2–4 years and >4 years of structured sprint training to examine whether training exposure influenced recovery stability.

Each subgroup analysis used the same primary outcomes: Recovery State and Recovery Index. SRA was not retrained separately for every subgroup during the main analysis; instead, subgroup performance was examined using the held-out predictions generated from the same validation structure. This procedure allowed subgroup interpretation without inflating model performance through repeated re-optimization. Subgroup comparisons focused on recovery-state classification consistency, return-to-baseline trajectory, and model error distribution across event specialty, sex, performance level, and training age.

### 2.5 Statistical analysis

All analyses were conducted using R (R Foundation for Statistical Computing, Austria). Participant characteristics, sprint background, and baseline performance were summarized using means and standard deviations for normally distributed continuous variables, medians and interquartile ranges for skewed variables, and frequencies with percentages for categorical variables. Normality was examined using Shapiro–Wilk tests and Q–Q plots, variance homogeneity using Levene’s test, and influential observations using standardized residuals. Recovery State was analyzed as a categorical primary outcome using mixed-effects multinomial logistic regression with athlete identifier as a random effect and testing interval as the fixed repeated factor. Recovery Index was analyzed as a continuous primary outcome using linear mixed-effects modeling, with testing interval as the main fixed effect and baseline sprint time, sex, event specialty, and training age entered as covariates.

The mixed-effects structure accounted for repeated observations nested within athletes. Athlete identifier was specified as a random intercept because each participant contributed baseline, post-fatigue, and repeated recovery measurements. Random slopes for testing interval were examined using likelihood-ratio comparison and Akaike information criterion and were retained only when model fit improved by ≥2 Akaike information criterion units without singular convergence. Fixed-effect estimates are reported with 95% confidence intervals and odds ratios for categorical recovery-state models or standardized regression coefficients for continuous outcomes.

Secondary outcomes were analyzed according to measurement type. Sprint Time and Jump Height were examined using repeated-measures multivariate analysis of variance across baseline, immediate post-fatigue, and recovery intervals. IMU feature-domain associations with Recovery Index were examined using repeated-measures principal component regression. Features were standardized within the analysis pipeline, principal components explaining at least 85% of cumulative variance were retained, and component scores were entered into a linear mixed-effects regression with athlete identifier as a random intercept. Principal-component extraction for predictive modeling was fitted exclusively within development-cohort training folds and transferred unchanged to validation observations. Exertion Score was treated as ordinal and analyzed using the Friedman test across testing intervals. SRA was compared with fixed models using held-out athlete predictions; paired recovery-state decisions were assessed using McNemar tests, while continuous prediction errors were compared using Wilcoxon signed-rank tests. Subgroup effects for event specialty, sex, performance level, and training age were examined through interaction terms in the primary recovery models.

Multiplicity across planned post-fatigue interval contrasts was controlled using the Holm procedure within each outcome family. Effect sizes included partial η² for repeated-measures multivariate comparisons, standardized β coefficients for continuous recovery models, odds ratios for categorical models, and paired standardized mean differences for interval contrasts. Model-performance uncertainty was quantified using 2,000 athlete-cluster bootstrap resamples to obtain 95% confidence intervals for accuracy, precision, recall, F1-score, area under the curve, mean absolute error, root mean square error, and pairwise performance differences. McNemar tests were applied to paired classification decisions, while paired bootstrap confidence intervals and Wilcoxon signed-rank tests were used for continuous prediction-error comparisons. IMU trials with >5% signal loss, synchronization mismatch >10 ms, or confirmed sensor displacement were treated as invalid and were not imputed. Intermittent missing scalar covariates below 5% were handled using multiple imputation by predictive mean matching with 20 imputations; analyses with complete data were retained as sensitivity checks. Fatigue-sprint count was included as a centered covariate to account for between-athlete variation in fatigue exposure. Model interpretability was evaluated using SHapley Additive exPlanations (SHAP) for held-out predictions; mean absolute SHAP values were aggregated by feature domain and normalized to 100% to quantify relative contribution to recovery-state classification. All statistical tests were two-tailed, with significance set at p< 0.05.

## 3 Results

### 3.1 Baseline features

Baseline sprint-event profiles establish the reference structure for interpreting recovery because the 100 m and 200 m groups were used as the main baseline grouping factor before SRA recovery estimation.

The baseline profile showed a balanced collegiate sprint sample across the two event specialties. The 100 m group included 66 athletes and the 200 m group included 58 athletes, with comparable age, sex distribution, training age, and weekly sprint-training frequency. Sprint-event specificity was visible in personal-best times, while baseline acceleration-phase performance remained similar, with 30 m sprint time recorded as 4.17 ± 0.18 s for 100 m athletes and 4.20 ± 0.19 s for 200 m athletes (**Table 1**). Baseline IMU profiles also showed closely aligned movement-control indices across event groups, including acceleration consistency above 90 in both groups and limb symmetry above 91 in both groups (**Table 1**). This comparability supports the use of athlete-specific baseline profiles in SRA because recovery interpretation depended on within-athlete deviation from stable non-fatigued mechanics, not on unequal starting movement quality between sprint-event groups.

**Table 1:** Baseline athlete and IMU profile by sprint event.

Baseline feature	100 m	200 m
Age (years)	20.42 ± 1.31	20.67 ± 1.28
Sex	Male: 38 (57.58%); Female: 28 (42.42%)	Male: 33 (56.90%); Female: 25 (43.10%)
Training age (years)	4.36 ± 1.21	4.58 ± 1.34
Weekly sprint training (sessions/week)	5.18 ± 0.74	5.31 ± 0.69
Personal best (s)	11.42 ± 0.48	23.20 ± 0.82
Baseline 30 m time (s)	4.17 ± 0.18	4.20 ± 0.19
Baseline 60 m time (s)	7.22 ± 0.29	7.30 ± 0.31
Jump height (cm)	51.60 ± 6.80	50.10 ± 7.20
Exertion Score	9.00 (8.00–10.00)	9.00 (8.00–10.00)
Stride variability (%)	3.82 ± 0.76	3.95 ± 0.81
Stride frequency (strides/s)	4.38 ± 0.29	4.31 ± 0.27
Acceleration consistency	91.40 ± 4.60	90.70 ± 4.90
Angular control	88.60 ± 5.30	87.90 ± 5.50
Trunk stability	89.30 ± 4.80	88.40 ± 5.10
Limb symmetry	92.10 ± 3.90	91.50 ± 4.20

The 100 m group included 66 athletes, and the 200 m group included 58 athletes. Acceleration consistency, angular control, trunk stability, and limb symmetry are baseline IMU-derived indices scaled from 0 to 100, with higher values indicating stronger baseline movement control.

Athlete-level allocation produced a 99-athlete development cohort and a 25-athlete held-out cohort. Mean age was 20.54 ± 1.30 and 20.56 ± 1.31 years, training age was 4.45 ± 1.27 and 4.52 ± 1.29 years, baseline 30 m time was 4.18 ± 0.18 and 4.20 ± 0.19 s, and jump height was 50.90 ± 6.91 and 50.52 ± 7.08 cm, respectively. The development cohort included 57 males and 42 females and the held-out cohort 14 males and 11 females. No material baseline differences were detected between cohorts across demographic, sprint-performance, or IMU characteristics (all p > 0.30).

### 3.2 Primary outcomes

Recovery State and Recovery Index answer RQ3 by characterizing individualized sprint-kinematic recovery across the predefined post-fatigue intervals. H3 is evaluated through the progressive increase in Recovery Index as IMU-derived sprint mechanics approach each athlete’s rested baseline profile.

SRA classified the predictor-independent protocol labels most consistently at baseline and immediate post-fatigue, with correct classification of 24/25 and 23/25 held-out observations, respectively (**Table 2**; **Figure 3A**). Classification was less distinct during transitional recovery, reaching 79.2% at 5 min, 83.3% at 10 min, 87.5% at 15 min, and 80.0% at 20 min (**Table 2**; **Figure 3A**). Errors occurred primarily between adjacent recovery categories, particularly early versus intermediate and intermediate versus late recovery, indicating overlap in movement characteristics during the post-fatigue trajectory rather than separation into sharply discrete mechanical states. Recovery Index increased from 54.20 ± 8.30 immediately post-fatigue to 86.80 ± 6.40 at 20 min (**Table 3**; **Figure 3B**). The mixed-effects interval effect was significant, F(4, 482.6) = 181.7, p< 0.001, partial η² = 0.60, and the 20 min contrast remained significant after Holm correction (MD = 32.60, 95% CI 29.61–35.59, adjusted p = 0.008, standardized effect = 2.21) (**Table 3**).

**Table 2:** Held-out SRA classification against protocol-defined recovery labels.

Assessment interval	Protocol label	n	Baseline	Acute fatigue	Early/intermediate/late recovery	Correct n (%)	Misclassified n (%)
Baseline	Baseline	25	24	0	1/0/0	24 (96.0%)	1 (4.0%)
Immediate post-fatigue	Acute fatigue	25	0	23	2/0/0	23 (92.0%)	2 (8.0%)
5 min	Early recovery	24	0	2	19/3/0	19 (79.2%)	5 (20.8%)
10 min	Intermediate recovery	24	0	0	3/20/1	20 (83.3%)	4 (16.7%)
15 min	Intermediate recovery	24	0	0	1/21/2	21 (87.5%)	3 (12.5%)
20 min	Late recovery	25	0	0	0/5/20	20 (80.0%)	5 (20.0%)

Values are n or n (%); 147 valid observations from 25 held-out athletes were analyzed. Three records with >5% IMU signal loss were excluded; protocol labels were predictor-independent.

**Figure 3:**
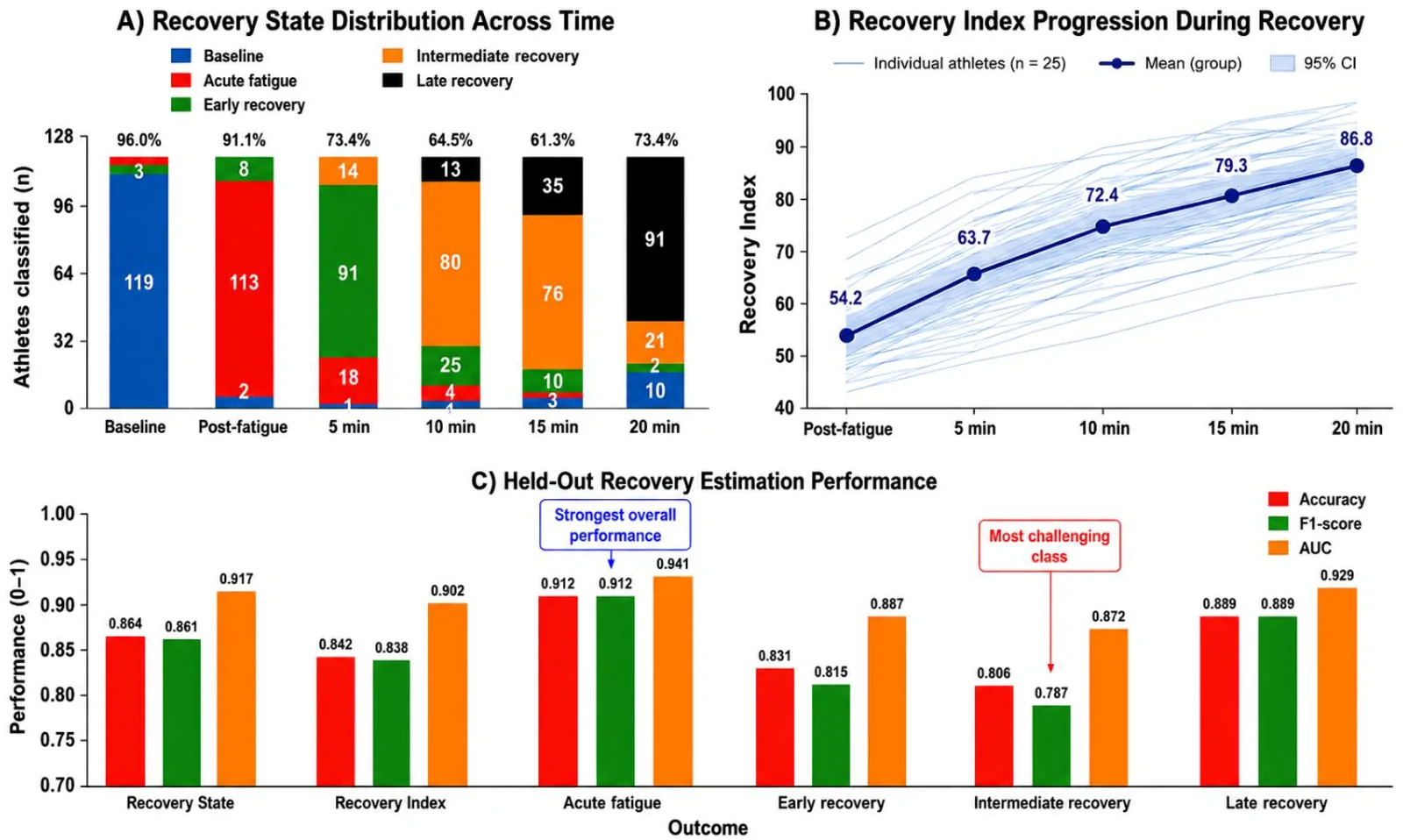
**(A)** SRA recovery-state distributions across baseline, post-fatigue, and recovery intervals; **(B)** Recovery Index progression with error bars and significance contrasts across post-fatigue recovery time points; and **(C)** held-out SRA performance for recovery-state and recovery-index estimation using accuracy, F1-score, and AUC.

**Table 3:** Recovery index across post-fatigue intervals.

Interval	Recovery index	MD#	SE	95% CI	Holm-adjusted p	Effect size
Immediate post-fatigue	54.20 ± 8.30	0.00	—	—	—	—
5 min	63.70 ± 8.10	9.50	1.18	7.16, 11.84	0.016	0.74
10 min	72.40 ± 7.60	18.20	1.34	15.55, 20.85	0.016	1.29
15 min	79.30 ± 7.10	25.10	1.42	22.29, 27.91	0.012	1.78
20 min	86.80 ± 6.40	32.60	1.51	29.61, 35.59	0.008	2.21

MD compares each interval with immediate post-fatigue. Estimates are from the linear mixed-effects model; p values are Holm-adjusted.

Individual Recovery Index trajectories showed substantial between-athlete variation despite the overall upward pattern, with immediate-to-20 min improvement ranging from approximately 18.4 to 46.7 points and a median improvement of 32.1 points (IQR 27.8–36.9) (**Figure 3B**). Nine of 25 held-out athletes satisfied the independent mechanical-recovery criteria by 15 min, increasing to 18 of 25 by 20 min.

Held-out recovery estimation supported the primary outcome because SRA maintained strong discrimination on completely unseen athlete data across the predefined recovery intervals. Recovery State classification reached 0.864 accuracy (95% CI 0.817–0.905), 0.861 F1-score (95% CI 0.812–0.902), and 0.917 AUC (95% CI 0.884–0.945), while Recovery Index estimation yielded MAE = 4.82 and RMSE = 6.21 (**Supplementary Table S2**; **Figure 3C**). Class-specific sensitivity ranged from 0.792 for early recovery to 0.960 for baseline, with intermediate and late recovery producing F1-scores of 0.845 and 0.833, respectively (**Table 2**; **Supplementary Table S2**). Calibration remained stable, with expected calibration error = 0.041, Brier score = 0.118, and calibration slope = 0.96 (**Supplementary Table S2**). Misclassification occurred mainly between adjacent transitional intervals, indicating greater overlap during intermediate recovery while baseline and acute-fatigue observations retained the clearest classification profiles in held-out evaluation.

### 3.3 Secondary outcomes

Sprint performance and IMU mechanics answer RQ1 by identifying which variables were most sensitive to fatigue and recovery. H1 is tested through changes in stride, acceleration, angular-velocity, stability, and symmetry features across the fatigue-recovery sequence.

Sprint-performance indicators confirmed that the fatigue protocol produced measurable disruption before recovery progressed. The 30 m sprint time increased from 4.18 ± 0.19 s at baseline to 4.48 ± 0.24 s post-fatigue, while jump height declined from 50.90 ± 6.95 cm to 44.60 ± 6.72 cm, indicating simultaneous impairment in acceleration performance and explosive lower-limb output (**Table 4**). Exertion Score also rose sharply after fatigue and then declined toward the 20 min interval, supporting the physiological plausibility of the recovery sequence (**Supplementary Table S3**; **Figure 4**). IMU sprint mechanics showed broader movement-control disruption, with acceleration consistency decreasing from 91.07 ± 4.74 at baseline to 73.60 ± 6.85 post-fatigue and angular control declining from 88.27 ± 5.40 to 70.80 ± 7.12. These changes indicate that fatigue affected both propulsion-related consistency and segmental rotational control during sprint movement.

**Table 4:** Sprint performance and exertion across time.

Outcome	Baseline	Post-fatigue	5 min	10 min	15 min	20 min	MD post-fatigue	MD 20 min	95% CI	Sig.
30 m time (s)	4.18 ± 0.19	4.48 ± 0.24	4.39 ± 0.23	4.31 ± 0.21	4.26 ± 0.20	4.22 ± 0.19	0.30	0.04	0.25, 0.35	0.006
Jump height (cm)	50.90 ± 6.95	44.60 ± 6.72	46.80 ± 6.65	48.20 ± 6.51	49.30 ± 6.44	50.00 ± 6.38	-6.30	-0.90	-7.42, -5.18	0.009
Exertion Score	9.10 ± 1.05	17.40 ± 1.12	14.80 ± 1.18	12.90 ± 1.14	11.30 ± 1.09	10.20 ± 1.06	8.30	1.10	7.91, 8.69	0.004

MD values compare post-fatigue and 20 min values with baseline.

**Figure 4:**
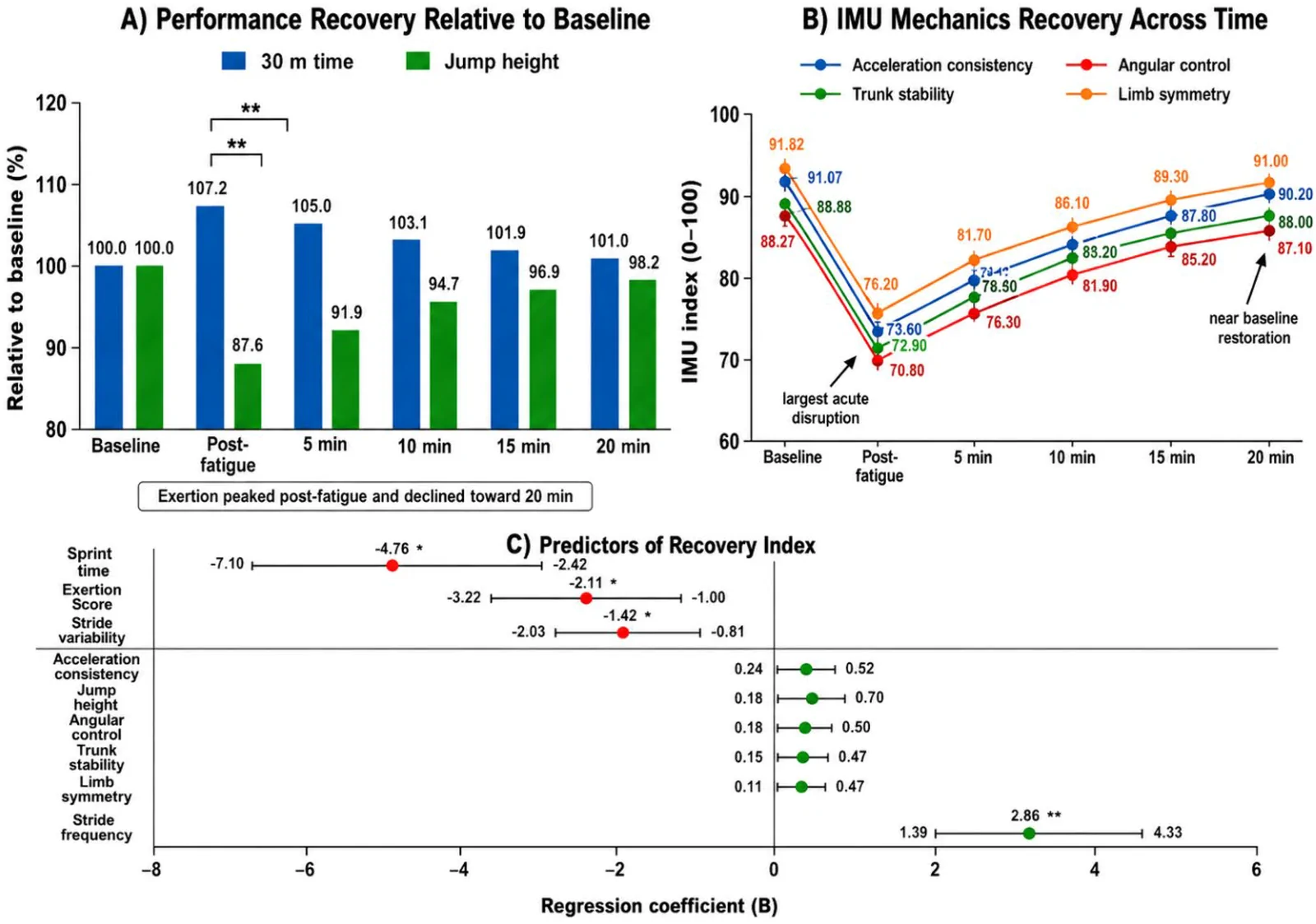
**(A)** Sprint performance recovery relative to baseline for 30 m time and jump height; **(B)** IMU mechanics recovery across baseline, post-fatigue, and recovery intervals for acceleration consistency, angular control, trunk stability, and limb symmetry; and **(C)** predictors of Recovery Index based on regression coefficients with 95% CI.

Athletes completed 7.9 ± 1.2 fatigue-induction sprints, with 45 completing 6–7 repetitions, 39 completing 8 repetitions, and 40 completing 9–10 repetitions; final-sprint slowing averaged 6.8 ± 1.4% relative to baseline. Sprint count showed a small association with immediate post-fatigue Recovery Index (β = −0.09, 95% CI −0.19–0.03, p = 0.151), and the sprint-count-by-interval interaction was not significant (p = 0.184), indicating a similar recovery trajectory across fatigue-dose strata (**Supplementary Table S3**).

Feature association analysis identified the IMU and performance variables most closely linked with individualized return-to-baseline recovery. Acceleration consistency showed the strongest positive standardized association with Recovery Index (β = 0.41, 95% CI 0.24, 0.52, p = 0.006), while angular control, trunk stability, and limb symmetry also contributed positively to recovery progression (**Supplementary Table S4**). Sprint time showed a negative association with Recovery Index (B = -4.76, 95% CI -7.10, -2.42, p = 0.019), indicating that slower sprint performance corresponded with poorer return-to-baseline movement status. These associations clarify why SRA used multiple movement domains instead of a single performance marker. Sprint recovery was reflected through integrated restoration of acceleration, rotational control, proximal stability, and bilateral coordination. The secondary outcomes therefore show that fatigue-sensitive IMU features carried recovery information beyond sprint time and jump height alone.

Classification interpretability produced a similar feature hierarchy to the Recovery Index weighting scheme (**Supplementary Table S4**). Normalized mean absolute SHAP contribution was 31.8% for acceleration consistency, 25.6% for angular-velocity control, 22.4% for trunk-pelvis stability, and 20.2% for inter-limb symmetry, corresponding closely with Recovery Index weights of 0.32, 0.26, 0.23, and 0.19, respectively (**Supplementary Table S4**). Recovery State was the prediction target and was not entered as a predictor. Acceleration consistency therefore contributed the largest share of held-out classification information, while all four movement domains retained measurable model influence.

### 3.4 Model robustness and transfer analyses

Algorithm comparison, ablation testing, cross-task feature-generalization testing, and subgroup analysis answer RQ2 by evaluating whether adaptive modeling improved individualized sprint-kinematic recovery estimation.

SRA outperformed the fixed comparison models on held-out athlete data, reaching 0.861 F1-score and 0.917 AUC and exceeding the strongest fixed comparator, convolutional neural network–long short-term memory, by ΔF1 = 0.039 (95% CI 0.014–0.064, p = 0.003) and ΔAUC = 0.028 (95% CI 0.011–0.047, p = 0.004) (**Supplementary Table S5**; **Figure 5A**). Ablation analysis showed that individualized baseline updating was the most influential adaptive component; its removal reduced F1-score from 0.861 to 0.769 (Δ = −0.092, 95% CI −0.124 to −0.061, p< 0.001) and increased RMSE from 6.21 to 8.72 (Δ = 2.51, 95% CI 1.88–3.17, p< 0.001) (**Supplementary Table S6**; **Figure 5B**). Temporal updating also produced a marked performance decline when removed, while threshold sensitivity remained stable at ±3%, ± 5%, and ±7%, with F1-scores of 0.852, 0.861, and 0.858, respectively (**Supplementary Table S6**). These results indicate that SRA performance depended principally on individualized baseline adaptation and temporal recovery information while remaining robust to reasonable variation in the recovery threshold.

**Figure 5:**
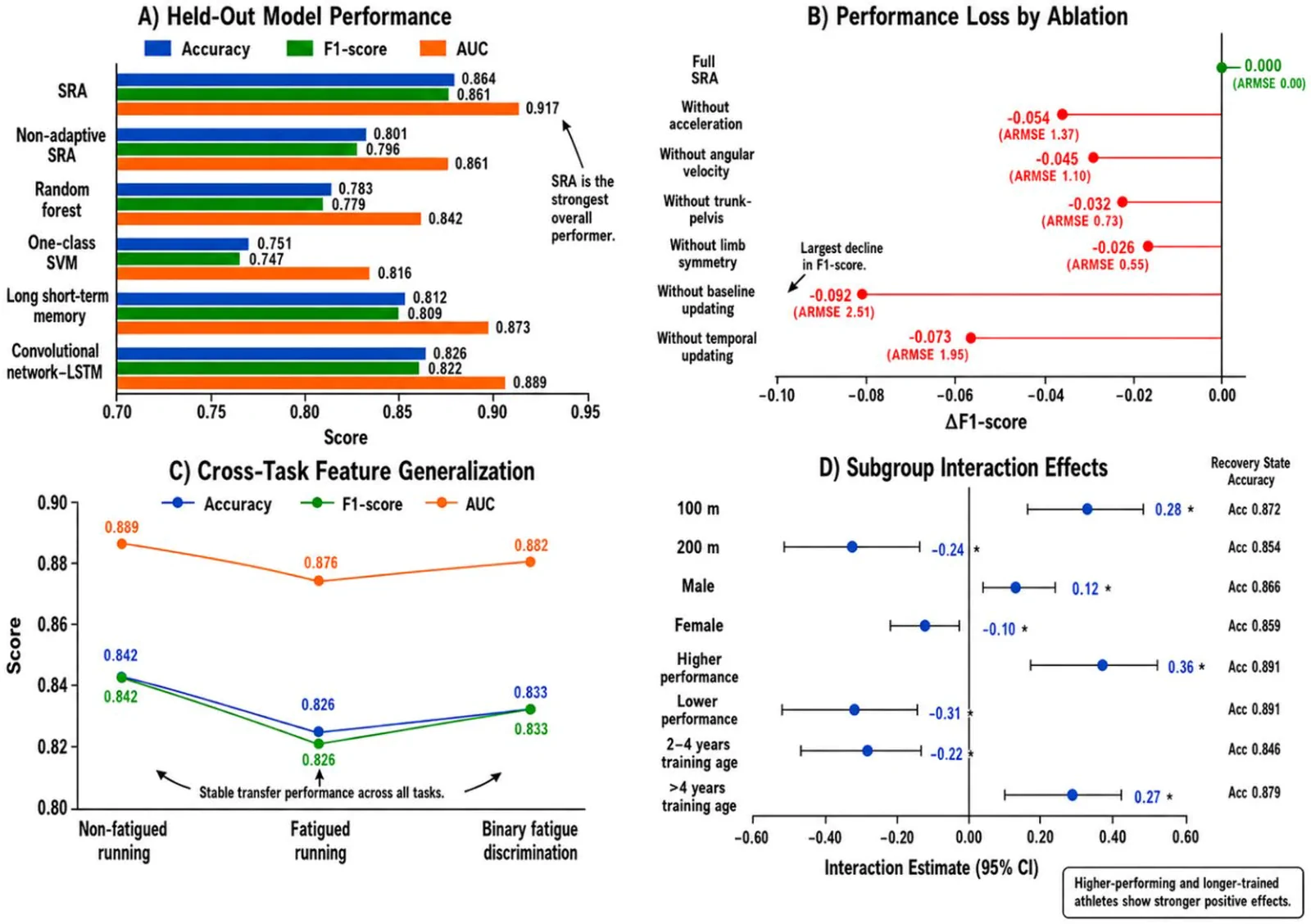
**(A)** Held-out SRA and baseline model performance using accuracy, F1-score, and AUC; **(B)** SRA ablation effects showing performance loss after removing key model components; **(C)** cross-task feature generalization for binary fatigued versus non-fatigued running discrimination; and **(D)** subgroup interaction effects with 95%CI and recovery-state accuracy across sprint-event, sex, performance, and training-age groups.

Cross-task feature-generalization testing yielded 0.833 accuracy, 0.833 F1-score, and 0.882 AUC for binary fatigued versus non-fatigued endurance-running discrimination after sensor-channel harmonization and application of the frozen feature transformations (**Supplementary Table S7**; **Figure 5C**). These values indicate that the SRA feature-extraction and fatigue-discrimination pipeline retained transferable discriminatory information across a distinct running task. Subgroup analysis showed stronger model consistency among higher-performing athletes, who recorded 0.891 recovery-state accuracy and a positive interaction estimate, while lower-performing athletes showed reduced accuracy and a negative interaction estimate (**Supplementary Table S8**). Longer-trained athletes also showed stronger positive subgroup effects than athletes with shorter training age. These subgroup patterns suggest that stable sprint mechanics and training exposure may make recovery trajectories more consistent and more learnable. The robustness analyses therefore support the internal adaptive contribution of SRA, stable held-out performance, cross-task portability of selected feature-processing operations, and subgroup-sensitive variation within the present collegiate sprint sample.

## 4 Discussion

The observed post-fatigue pattern showed a progressive return of sprint performance and IMU-derived movement measures toward athlete-specific baseline values across the 20 min recovery window. This trajectory answers RQ3 by showing that SRA differentiated protocol-defined assessment intervals and quantified individualized sprint-kinematic recovery in held-out athletes, while H3 was supported by the progressive Recovery Index increase and held-out classification performance. The observed changes in sprint time, jump capacity, acceleration consistency, angular-velocity control, trunk stability, and limb symmetry answer RQ1 because they identify the IMU-derived sprint-biomechanical features most sensitive to fatigue and recovery. These movement changes also support H1, as stride, acceleration, rotational-control, and stability features shifted across baseline, post-fatigue, and recovery states in a direction consistent with fatigue-induced mechanical disruption. The comparison between SRA and fixed machine-learning models answers RQ2 and supports H2, because adaptive modeling produced stronger individualized recovery estimation than fixed alternatives. The analytical contribution of SRA was supported by its conversion of multidomain IMU signals into athlete-specific recovery estimates through feature extraction, individualized baseline referencing, and temporal updating. The strongest contribution came from baseline updating and temporal recovery updating, indicating that recovery monitoring becomes more informative when each athlete’s fatigued movement pattern is interpreted against their own non-fatigued sprint profile.

Across the held-out cohort, Recovery Index increased from 54.20 ± 8.30 immediately post-fatigue to 86.80 ± 6.40 at 20 min, while recovery-state classification reached 0.864 accuracy, 0.861 F1-score, and 0.917 AUC. Individual trajectories varied substantially around the group progression, with only 9 of 25 athletes meeting the independent mechanical-recovery criteria by 15 min and 18 of 25 by 20 min. The combination of increasing group-level Recovery Index values, heterogeneous athlete trajectories, and greater classification overlap between adjacent recovery intervals supports interpretation of SRA as an individualized recovery-trajectory estimator.

The analysis suggests that individualized adaptive modeling provided a stronger basis for interpreting sprint fatigue recovery than fixed model estimation, especially because recovery was anchored to each athlete’s own non-fatigued movement profile. Biró et al. (2026) similarly reported that personalized fatigue-detection models performed more effectively than population-level models, showing that neuromuscular fatigue signatures vary across athletes and require athlete-specific interpretation. The present study extends that logic to post-fatigue trajectory estimation, where individualized baseline updating contributed substantially to discrimination of movement patterns at different stages of return toward baseline. Marotta et al. (2021) also showed that IMU-based fatigue classification in real-world track running depended strongly on sensor configuration and fatigue-sensitive variables such as acceleration magnitude, angular velocity, and gait variability. This aligns with the current pattern, where acceleration consistency, angular-velocity control, trunk stability, and limb symmetry operated as meaningful recovery markers after repeated sprint loading. The contribution of SRA is therefore its shift from general fatigue recognition toward recovery interpretation across time. Its feature-extraction layer captured sprint-specific mechanical disruption, while individualized profiling prevented recovery estimates from being judged against a generic athlete profile. Adaptive estimation combined the magnitude of movement deviation with preceding recovery information to characterize progression across the protocol-defined intervals. The resulting estimates therefore describe changes in sprint-kinematic proximity to baseline within the observed 20 min period.

The ablation findings require interpretation at the computational level. Removal of individualized baseline updating reduced F1-score from 0.861 to 0.769 (ΔF1 = −0.092), while removal of temporal updating also produced a substantial performance loss. Baseline updating changes both the athlete-specific reference information available to the model and the adaptive computation applied to that information, so the observed decline cannot isolate a single biological mechanism. The result supports the predictive value of individualized reference information and temporal history within SRA, while controlled variants that preserve equivalent information content would be required to separate their individual computational mechanisms.

Held-out classification was strongest for baseline and acute-fatigue observations, while greater overlap occurred between adjacent early, intermediate, and late recovery intervals. Chang et al. (2023) reported that inertial-sensor time series, heart-rate response, perceived exertion, and hybrid deep-learning architectures could separate pre-fatigue, mid-fatigue, and post-fatigue running states, which supports the present use of staged recovery labels after repeated sprint loading. The current study applies staged classification to a post-fatigue sequence in which IMU-derived movement characteristics progressively approached each athlete’s baseline profile. Biró et al. (2023) showed that multivariate IMU time-series patterns can reveal subtle fatigue-related movement changes before visual detection, aligning with the current finding that wearable signals captured recovery transitions across post-fatigue intervals. SRA strengthened this process by combining temporal movement information with athlete-specific recovery profiling. Acute fatigue was easier to identify because mechanical disruption was concentrated and directionally consistent across sprint performance, acceleration control, and trunk-limb coordination. Later intervals showed greater classification overlap and between-athlete variation as different movement domains approached baseline at different rates. This pattern supports analysis of recovery as a dynamic movement trajectory across repeated post-fatigue observations.

The observed pattern was characterized by concurrent changes in acceleration consistency, angular-velocity control, trunk stability, and limb symmetry, with each domain approaching athlete-specific baseline values at a different rate. Dimmick et al. (2023) reported that inertial-sensor fatigue monitoring captured performance decline through torso-pelvis adaptations and strong inter-individual variation, which aligns with the current emphasis on athlete-specific recovery profiling. Mitschke et al. (2025) found that fatigue appeared more clearly in tibial acceleration, foot angular velocity, and rearfoot movement than in basic stride descriptors, supporting the present use of acceleration and rotational-control features as sensitive recovery markers. Within the present dataset, the same movement domains showed parallel but non-identical post-fatigue trajectories, supporting multidomain characterization of sprint-kinematic recovery. SRA strengthens this interpretation because its baseline-referenced design allows the same mechanical signal to be judged according to the athlete’s own sprint profile. Acceleration features captured the immediate loss of propulsive control, angular-velocity features reflected rotational disruption in the foot-shank-pelvis chain, and trunk-pelvis stability indicated whether proximal control had normalized. Acceleration consistency showed the largest normalized SHAP contribution at 31.8%, followed by angular-velocity control at 25.6%, trunk-pelvis stability at 22.4%, and limb symmetry at 20.2%. This ordering closely corresponded with Recovery Index weights of 0.32, 0.26, 0.23, and 0.19, respectively. The agreement between regression associations, Recovery Index weighting, and held-out SHAP contributions indicates that classification information was distributed across several movement domains, with acceleration consistency contributing the largest individual share.

Subgroup results indicate possible heterogeneity in model performance but require conservative interpretation because the held-out strata were small. The held-out cohort contained 13–100 m and 12–200 m athletes, 14 males and 11 females, approximately 8 athletes in each performance tertile, and 14 athletes with 2–4 years versus 11 with >4 years of training. Higher-performing athletes showed 0.891 recovery-state accuracy and a positive interaction estimate of 0.36, while lower-performing athletes showed 0.831 accuracy and an estimate of −0.31; longer-trained athletes similarly showed a positive estimate of 0.27. The accompanying 95% confidence intervals indicate uncertainty around these subgroup effects, and differences may reflect movement consistency, signal characteristics, or sample composition. Generalization to elite, youth, masters, or other athlete populations therefore requires independent evaluation.

### 4.1 Theoretical and practical implications

SRA refines fatigue-recovery modeling by shifting IMU analysis from static fatigue detection to individualized return-to-baseline estimation. The findings support an adaptive recovery logic in which acceleration consistency, angular-velocity control, trunk-pelvis stability, and limb symmetry operate as complementary indicators of short-term sprint-kinematic return toward baseline.

Sprint-training monitoring may use athlete-specific IMU deviations as supplementary information when assessing short-term movement recovery after repeated high-intensity sprint loading. The present findings support monitoring applications for recovery profiling and session review; real-time deployment, automated training prescription, clinical decision-making, and improvements in subsequent training outcomes were not evaluated.

### 4.2 Limitations and future research

The study focused on Chinese collegiate 100 m and 200 m sprinters, so subsequent studies should test SRA in elite sprinters, youth athletes, masters athletes, and athletes from different training systems. The recovery window ended at 20 min and therefore characterized only short-term changes in sprint performance and movement mechanics; longitudinal designs should extend monitoring across several hours and subsequent training days. Cross-task feature-generalization testing used an endurance-running IMU dataset because independent sprint-recovery datasets remain scarce; sprint-specific external validation is therefore still required before generalizing recovery-estimation performance beyond the present cohort. The model used IMU, sprint time, jump height, and exertion indicators, while biochemical, sleep, and muscle-damage markers were not included; expanded sampling should integrate physiological markers to examine whether SRA reflects broader recovery status beyond movement mechanics.

No independent physiological reference such as electromyography, blood biomarkers, direct muscle-function testing, or other neuromuscular measurements was available, so the observed trajectory is limited to sprint-kinematic and performance-related recovery. Baseline testing involved three 60 m maximal sprints whereas recovery monitoring used one 30 m maximal-effort trial at each interval; matched 0–30 m acceleration segments reduced this task difference, although differences in trial number and accumulated loading remain potential sources of measurement variation. Real-time deployment, long-term recovery forecasting, and intervention-driven changes in training outcomes were outside the present validation design.

### 4.3 Conclusion

The study shows that IMU-derived sprint mechanics can differentiate protocol-defined post-fatigue intervals and quantify short-term return toward athlete-specific baseline profiles in collegiate 100 m and 200 m sprinters. SRA provided a structured analytical pathway linking feature extraction, athlete-specific baseline profiling, and adaptive recovery-trajectory estimation. The study identified fatigue-sensitive sprint-biomechanical features, demonstrated stronger held-out recovery estimation than fixed comparison models, and characterized progressive movement return across the predefined recovery intervals. The framework converts wearable sensor signals into athlete-specific short-term recovery information through integrated acceleration, rotational-control, trunk-pelvis, and symmetry features. These findings support further development of IMU-assisted short-term recovery monitoring for sprint training and university-level athlete management, with prospective deployment and independent physiological validation required before broader decision-support applications.

## Data Availability

The original contributions presented in the study are included in the article/**Supplementary Material**. Further inquiries can be directed to the corresponding author.
