# Supplementary material for: Dynamic monitoring and adaptive algorithm optimization of fatigue recovery in track and field athletes based on inertial measurement unit sensor data
Source: Front Physiol. 2026 Oct 6;17:1919336. doi: 10.3389/fphys.2026.1919336 (PMC13680242; doi:10.3389/fphys.2026.1919336)
Supplement: Supplementary file 1 [file DataSheet1.docx]

**Table S1: SRA architecture and operational parameters used for adaptive recovery-state estimation.**

| **SRA stage** | **Input class** | **Feature class** | **Operational computation** | **Parameter setting** | **Output class** |
| --- | --- | --- | --- | --- | --- |
| Stage 1: IMU feature extraction | Lumbar, pelvis, left shank, right shank, left foot, right foot | Acceleration consistency | Stride-to-stride coefficient of variation of resultant acceleration | 200 Hz sampling; ±16 g accelerometer range; stride window from foot-strike to next ipsilateral foot-strike | Athlete-level feature vector |
| Stage 1: IMU feature extraction | Foot and shank sensors | Angular-velocity control | Peak, mean, and variability of sagittal and transverse angular velocity | ±2000 °/s gyroscope range; accepted onset mismatch ≤10 ms | Lower-limb rotational-control score |
| Stage 1: IMU feature extraction | Lumbar and pelvis sensors | Trunk-pelvis stability | Root mean square and coefficient of variation of lumbar and pelvic acceleration | Low-pass filtering at 20 Hz; stride-level segmentation | Stability score |
| Stage 1: IMU feature extraction | Bilateral foot and shank sensors | Inter-limb symmetry | Absolute left–right percentage difference in matched stride features | Symmetry deviation accepted only from complete bilateral strides | Symmetry score |
| Stage 2: Individualized profiling | Rested baseline trials | Baseline control profile | Athlete-specific mean and standard deviation across valid baseline strides | Minimum 3 valid baseline trials; ≥6 valid strides per trial | Individual reference profile |
| Stage 3: Adaptive estimation | Post-fatigue and recovery trials | Return-to-baseline recovery index | Weighted distance between recovery features and baseline profile | Adaptive feature weights updated per athlete-session | Rested baseline, acute fatigue, early recovery, intermediate recovery, late recovery |

Note. Recovery-state classes were assigned from the same testing sequence for all athletes: rested baseline before fatigue, acute fatigue immediately after fatigue, early recovery at 5 min, intermediate recovery at 10–15 min, and late recovery at 20 min.

**Table S2: SRA recovery estimation in held-out athletes**

| **Outcome / class** | **Split** | **Accuracy** | **Sensitivity** | **Specificity** | **Precision** | **F1-score** | **AUC** | **Bootstrap 95% CI†** | **MAE** | **RMSE** |
| --- | --- | --- | --- | --- | --- | --- | --- | --- | --- | --- |
| Recovery State overall | Held-out | 0.864 | 0.864 | — | 0.858 | 0.861 | 0.917 | 0.812–0.902 | — | — |
| Recovery Index | Held-out | — | — | — | — | — | — | — | 4.82 | 6.21 |
| Baseline | Held-out | — | 0.960 | 1.000 | 1.000 | 0.980 | 0.971 | 0.914–1.000 | — | — |
| Acute fatigue | Held-out | — | 0.920 | 0.984 | 0.920 | 0.920 | 0.948 | 0.821–0.978 | — | — |
| Early recovery | Held-out | — | 0.792 | 0.943 | 0.731 | 0.760 | 0.887 | 0.641–0.858 | — | — |
| Intermediate recovery | Held-out | — | 0.854 | 0.919 | 0.837 | 0.845 | 0.895 | 0.761–0.909 | — | — |
| Late recovery | Held-out | — | 0.800 | 0.975 | 0.870 | 0.833 | 0.914 | 0.724–0.908 | — | — |

*Note.* †95% CI refers to F1-score. Overall calibration: ECE = 0.041, multiclass Brier score = 0.118, and calibration slope = 0.96 (95% CI 0.88–1.05).

**Table S3: IMU sprint mechanics across time**

| **Outcome** | **Baseline** | **Post-fatigue** | **5 min** | **10 min** | **15 min** | **20 min** | **MD post-fatigue vs baseline** | **MD 20 min vs baseline** | **95% CI** | **Holm-adjusted p** |
| --- | --- | --- | --- | --- | --- | --- | --- | --- | --- | --- |
| Stride variability (%) | 3.88 ± 0.79 | 6.72 ± 1.18 | 5.81 ± 1.09 | 5.02 ± 0.98 | 4.41 ± 0.88 | 4.03 ± 0.82 | 2.84 | 0.15 | 2.57, 3.11 | 0.018 |
| Stride frequency (strides/s) | 4.35 ± 0.28 | 4.02 ± 0.31 | 4.10 ± 0.30 | 4.19 ± 0.29 | 4.27 ± 0.28 | 4.32 ± 0.27 | −0.33 | −0.03 | −0.40, −0.26 | 0.024 |
| Acceleration consistency | 91.07 ± 4.74 | 73.60 ± 6.85 | 79.40 ± 6.22 | 84.10 ± 5.56 | 87.80 ± 5.02 | 90.20 ± 4.81 | −17.47 | −0.87 | −19.08, −15.86 | 0.014 |
| Angular control | 88.27 ± 5.40 | 70.80 ± 7.12 | 76.30 ± 6.64 | 81.90 ± 6.02 | 85.20 ± 5.58 | 87.10 ± 5.31 | −17.47 | −1.17 | −19.20, −15.74 | 0.016 |
| Trunk stability | 88.88 ± 4.96 | 72.90 ± 6.48 | 78.50 ± 6.11 | 83.20 ± 5.74 | 86.00 ± 5.28 | 88.00 ± 5.02 | −15.98 | −0.88 | −17.43, −14.53 | 0.020 |
| Limb symmetry | 91.82 ± 4.05 | 76.20 ± 6.03 | 81.70 ± 5.64 | 86.10 ± 5.12 | 89.30 ± 4.61 | 91.00 ± 4.18 | −15.62 | −0.82 | −17.01, −14.23 | 0.024 |

*Note.* MD values compare post-fatigue and 20 min observations with baseline; p values are Holm-adjusted.

**Table S4: IMU feature associations and SRA feature contributions**

| **Predictor** | **B** | **β** | **SE** | **t** | **95% CI** | **p** | **Recovery Index weight** | **SHAP contribution (%)** |
| --- | --- | --- | --- | --- | --- | --- | --- | --- |
| Stride variability | −1.42 | −0.29 | 0.31 | −4.58 | −2.03, −0.81 | 0.017 | — | — |
| Stride frequency | 2.86 | 0.24 | 0.74 | 3.86 | 1.39, 4.33 | 0.026 | — | — |
| Acceleration consistency | 0.38 | 0.41 | 0.07 | 5.43 | 0.24, 0.52 | 0.006 | 0.32 | 31.8 |
| Angular control | 0.34 | 0.37 | 0.08 | 4.25 | 0.18, 0.50 | 0.014 | 0.26 | 25.6 |
| Trunk stability | 0.31 | 0.33 | 0.08 | 3.88 | 0.15, 0.47 | 0.021 | 0.23 | 22.4 |
| Limb symmetry | 0.29 | 0.30 | 0.09 | 3.22 | 0.11, 0.47 | 0.033 | 0.19 | 20.2 |
| Sprint time | −4.76 | −0.35 | 1.18 | −4.03 | −7.10, −2.42 | 0.019 | — | — |
| Jump height | 0.44 | 0.28 | 0.13 | 3.38 | 0.18, 0.70 | 0.030 | — | — |
| Exertion Score | −2.11 | −0.32 | 0.56 | −3.77 | −3.22, −1.00 | 0.024 | — | — |

*Note.* Recovery Index weights sum to 1.00; SHAP values are normalized mean absolute contributions from held-out predictions.

**Table S5: SRA and baseline model performance**

| **Model** | **Split** | **Accuracy** | **Precision** | **Recall** | **F1-score** | **AUC** | **MAE** | **RMSE** | **Time (ms)** |
| --- | --- | --- | --- | --- | --- | --- | --- | --- | --- |
| SRA | Held-out | 0.864 | 0.858 | 0.864 | 0.861 | 0.917 | 4.82 | 6.21 | 18.40 |
| Non-adaptive SRA | Held-out | 0.801 | 0.792 | 0.801 | 0.796 | 0.861 | 6.38 | 8.02 | 14.70 |
| Random forest | Held-out | 0.783 | 0.776 | 0.783 | 0.779 | 0.842 | 6.91 | 8.55 | 12.60 |
| One-class support vector machine | Held-out | 0.751 | 0.744 | 0.751 | 0.747 | 0.816 | 7.44 | 9.18 | 10.80 |
| Long short-term memory | Held-out | 0.812 | 0.807 | 0.812 | 0.809 | 0.873 | 6.05 | 7.71 | 27.30 |
| Convolutional neural network–long short-term memory | Held-out | 0.826 | 0.819 | 0.826 | 0.822 | 0.889 | 5.71 | 7.29 | 31.60 |

Note. Higher accuracy, precision, recall, F1-score, and AUC indicate stronger recovery-state classification. Lower MAE, RMSE, and time indicate lower prediction error and faster inference.

**Table S6: SRA ablation performance**

| **Variant** | **Accuracy** | **F1-score** | **AUC** | **MAE** | **RMSE** | **ΔF1-score** | **ΔRMSE** |
| --- | --- | --- | --- | --- | --- | --- | --- |
| Full SRA | 0.864 | 0.861 | 0.917 | 4.82 | 6.21 | 0.000 | 0.00 |
| Without acceleration | 0.812 | 0.807 | 0.873 | 5.96 | 7.58 | -0.054 | 1.37 |
| Without angular velocity | 0.821 | 0.816 | 0.884 | 5.74 | 7.31 | -0.045 | 1.10 |
| Without trunk-pelvis | 0.833 | 0.829 | 0.891 | 5.43 | 6.94 | -0.032 | 0.73 |
| Without limb symmetry | 0.839 | 0.835 | 0.897 | 5.28 | 6.76 | -0.026 | 0.55 |
| Without baseline updating | 0.776 | 0.769 | 0.834 | 6.87 | 8.72 | -0.092 | 2.51 |
| Without temporal updating | 0.794 | 0.788 | 0.851 | 6.42 | 8.16 | -0.073 | 1.95 |

Note. ΔF1-score and ΔRMSE are calculated against full SRA. The largest decline occurred after removing individualized baseline updating, followed by temporal updating and acceleration features.

**Table S7: SRA transfer performance on external IMU running data**

| **Task** | **Sensor channels** | **Feature domains** | **Accuracy** | **Precision** | **Recall** | **F1-score** | **AUC** | **MAE** | **RMSE** |
| --- | --- | --- | --- | --- | --- | --- | --- | --- | --- |
| Non-fatigued running | Lumbar, shank, foot | Acceleration, angular velocity, symmetry | 0.842 | 0.851 | 0.834 | 0.842 | 0.889 | 5.26 | 6.68 |
| Fatigued running | Lumbar, shank, foot | Acceleration, angular velocity, stability | 0.824 | 0.813 | 0.839 | 0.826 | 0.876 | 5.73 | 7.14 |
| Binary fatigue discrimination | Lumbar, shank, foot | Acceleration, angular velocity, stability, symmetry | 0.833 | 0.829 | 0.837 | 0.833 | 0.882 | 5.49 | 6.91 |

Note. External validation used harmonized IMU running data to test SRA transfer beyond the sprint-specific sample. Performance was interpreted as fatigue discrimination robustness, not sprint-recovery classification.

**Table S8: SRA performance by athlete subgroup**

| **Subgroup** | **n** | **Recovery Index at 20 min** | **Recovery State accuracy** | **F1-score** | **AUC** | **RMSE** | **Interaction estimate** | **95%CI** | **Sig.** |
| --- | --- | --- | --- | --- | --- | --- | --- | --- | --- |
| 100 m | 66 | 87.60 ± 6.10 | 0.872 | 0.869 | 0.924 | 5.96 | 0.28 | 0.09, 0.47 | 0.032 |
| 200 m | 58 | 85.90 ± 6.70 | 0.854 | 0.849 | 0.909 | 6.48 | -0.24 | -0.43, -0.05 | 0.041 |
| Male | 71 | 87.10 ± 6.30 | 0.866 | 0.862 | 0.918 | 6.13 | 0.12 | 0.03, 0.21 | 0.044 |
| Female | 53 | 86.40 ± 6.60 | 0.859 | 0.855 | 0.913 | 6.32 | -0.10 | -0.19, -0.01 | 0.047 |
| Higher performance | 42 | 88.90 ± 5.70 | 0.891 | 0.887 | 0.936 | 5.54 | 0.36 | 0.15, 0.57 | 0.018 |
| Lower performance | 41 | 84.70 ± 6.90 | 0.831 | 0.825 | 0.894 | 6.92 | -0.31 | -0.52, -0.10 | 0.027 |
| 2–4 years training age | 59 | 85.60 ± 6.80 | 0.846 | 0.841 | 0.904 | 6.63 | -0.22 | -0.41, -0.03 | 0.039 |
| >4 years training age | 65 | 88.00 ± 5.90 | 0.879 | 0.875 | 0.928 | 5.88 | 0.27 | 0.08, 0.46 | 0.035 |

Note. Recovery Index values are presented as mean ± SD. Interaction estimates reflect subgroup differences in Recovery Index trajectory and recovery-state accuracy across the held-out prediction structure.

Figure S1: SRA computational pipeline for IMU feature processing, adaptive recovery estimation, model validation, and held-out athlete evaluation.
